# Sildenafil-Mediated Neuroprotection from Adult to Neonatal Brain Injury: Evidence, Mechanisms, and Future Translation

**DOI:** 10.3390/cells10102766

**Published:** 2021-10-15

**Authors:** Manuela Zinni, Julien Pansiot, Pierre-Louis Léger, Marina El Kamouh, Olivier Baud

**Affiliations:** 1 Inserm UMR1141 NeuroDiderot, Université de Paris, 75019 Paris, France; manuela.zinni@inserm.fr (M.Z.); julien.pansiot@inserm.fr (J.P.); marina.el-kamouh@inrae.fr (M.E.K.); 2 Pediatric and Neonatal Intensive Care Unit, Armand-Trousseau University Hospital, Assistance Publique-Hôpitaux de Paris, Sorbonne University, 75019 Paris, France; pierre-louis.leger@aphp.fr; 3 Laboratoire de Physiologie et Génomique des Poissons-INRAE, 35700 Rennes, France; 4 Laboratory of Child Growth and Development, University of Geneva, 1211 Geneva, Switzerland; 5 Division of Neonatology and Pediatric Intensive Care, Children’s University Hospital of Geneva, 1211 Geneva, Switzerland

**Keywords:** sildenafil, neuroprotection, brain injury

## Abstract

Cerebral stroke, traumatic brain injury, and hypoxic ischemic encephalopathy are among the most frequently occurring brain injuries. A complex pathogenesis, characterized by a synergistic interaction between alterations of the cerebrovascular system, cell death, and inflammation, is at the basis of the brain damage that leads to behavioral and neurodevelopmental disabilities in affected subjects. Sildenafil is a selective inhibitor of the enzyme phosphodiesterase 5 (PDE5) that is able to cross the blood–brain barrier. Preclinical data suggest that sildenafil may be a good candidate for the prevention or repair of brain injury in both adults and neonates. The aim of this review is to summarize the evidence supporting the neuroprotective action of sildenafil and discuss the possible benefits of the association of sildenafil with current therapeutic strategies.

## 1. Introduction

Cerebral stroke and traumatic brain injury (TBI) in adults and stroke and hypoxic ischemic encephalopathy (HIE) in neonates are among the most frequently occurring brain injuries recorded worldwide [1,2,3]. Despite the difference in developmental stage at the time of insult, these pathologies are all characterized by similarities in alterations of brain physiology and function. The brain damage and subsequent behavioral disorders and neurodevelopmental impairment are the result of a complex pathogenesis, defined by a synergistic interaction between alterations of the cerebrovascular system, cell death, and inflammation. Therapeutic strategies targeting cerebrovascular system, cell death, and the brain inflammatory response may represent a plausible therapeutic approach. Sildenafil is a selective inhibitor of the enzyme phosphodiesterase 5 (PDE5) and studies performed on animal models have demonstrated its ability to modulate these processes in the context of adult and neonatal brain injury and disease. The aim of this review is to summarize the evidence supporting the protective action of sildenafil in the developing and mature brain.

## 2. Materials and Methods

A literature search was performed in September 2021 using the Pubmed and Google Scholar databases in English without any restriction of the year, species, or authors. The following keywords were used in title (both Pubmed and Google Scholar search) or in abstract (Pubmed search): sildenafil or PDE5 inhibitor and stroke (121 articles), sildenafil or PDE5 inhibitor and traumatic brain injury (6 articles), sildenafil PDE5 inhibitor and hypoxic ischemic encephalopathy (1 article), sildenafil PDE5 inhibitor and neuroinflammation (24 articles), sildenafil PDE5 inhibitor and cerebral blood flow (28 articles), sildenafil PDE5 inhibitor and cell death (40 articles). An additional manual search was performed based on the references included in the selected research papers. Review papers were used as references only for general concepts. Only papers that met the following criteria were included: pertinence to the subject, presence of control groups, and clear descriptions of experimental procedures.

## 3. Nitric Oxide Pathway and Sildenafil: Key Modulators of Cerebral Blood Flow (CBF)

### 3.1. Nitric Oxide and CBF

The role of CBF in the pathogenesis of both adult and neonatal brain injury and disease has been clearly established. Restoration of the cerebral blood supply could thus represent a plausible therapeutic option for such brain injuries. In this context, the modulation of the nitric oxide (NO) pathway may represent a relevant target [4]. 

Preclinical pharmacological studies have shown a direct link between CBF and NO signaling. In vivo administration of L-NAME (NOS inhibitor) reduces cortical and hypothalamic CBF and increases the arterial blood pressure in rats, mice and lambs [5,6,7,8]. Coumans et al. and Hunter et al. demonstrated that L-NAME administration prevents the increase of CBF induced by exposure to hypoxia in preterm and term sheep [5,9]. Consistent with these observations, a study performed on endothelial NOS (eNOS) KO mice showed the reduction of CBF in response to TBI to be greater in mutant mice than in WT animals [10]. 

A strategy to increase NO levels and consequently restore and increase CBF is represented by the inhalation of NO. Inhaled nitric oxide (iNO), approved by the US Food and Drug Administration (FDA) for the treatment of persistent pulmonary hypertension in newborns [11], was initially studied in the context of brain stroke by Terpolilli et al. in 2012. The authors demonstrated, for the first time, the ability of iNO to reach the brain vasculature and directly induce cerebral venodilation when administrated at a dose of 50 ppm to healthy mice. Starting from this initial observation, Terpolilli et al. used an elegant multispecies approach to show that 50 ppm iNO induces venule and arteriole dilation and significantly increases perfusion of the penumbra in a mouse model of neonatal HIE and in mouse and sheep stroke models. The increased collateral CBF induced by NO inhalation was associated with a significant reduction in ischemic brain damage [12]. The protective effect of NO was confirmed by successive studies in preclinical models of neonatal ischemia in rats that showed a significant reduction of lesion size 72 h (50 ppm iNO) and 48 h (20 ppm iNO) after the induction of stroke and an increase in blood flow velocity [13,14]. 

Despite the beneficial effects observed in preclinical studies, the use of iNO in the context of brain injury presents several limitations, including dosage and the window of exposure. 

### 3.2. Phosphodiesterases (PDEs) and CBF: A Key Role for Sildenafil

Another strategy to modulate NO pathway is to control cyclic guanosine-3′,5′-monophosphate (cGMP) concentration, that mediates most of the biological effects of NO [15]. The enzyme soluble guanylyl cyclase (sGC) is the major physiological NO receptor in the brain and its activation occurs via a conformational change upon the binding of NO to the prosthetic heme, after which it catalyzes the conversion of GTP to cGMP. The increase in the intracellular cGMP concentration directly mediates the effects of NO and induces the activation of downstream cGMP-dependent protein kinases and cGMP-gated ion channels [16,17,18,19]. 

The stability of cGMP is a critical regulator of NO signal transduction and is dependent on the activity of phosphodiesterases (PDEs), of which 11 mammalian isoforms have been described. PDEs can be classified into three groups depending on their specificity: (i) cyclic AMP-specific (PDE4, PDE7, and PDE8), (ii) cyclic GMP-specific (PDE5, PDE6, and PDE9), (iii) and those that hydrolyze both cyclic GMP and AMP (PDE1, PDE2, PDE3, PDE10, and PDE11). Almost all PDE isoforms are expressed in the brain [20,21]. Due to their key role in the modulation of NO signaling, the inhibition of PDE activity to prolong the half-life of cGMP could represent a second therapeutic strategy to enhance the effect of endogenous NO and its effect on CBF in the context of brain injury. 

In particular, PDE5 is a GMP-specific PDE that is highly expressed in vascular smooth cell (VSMCs) and among the most abundant in arterial smooth cells (SMCs) [22]. Its expression has been reported in cerebral and meningeal arteries, both in humans and rodents [23].

**Figure 1:**
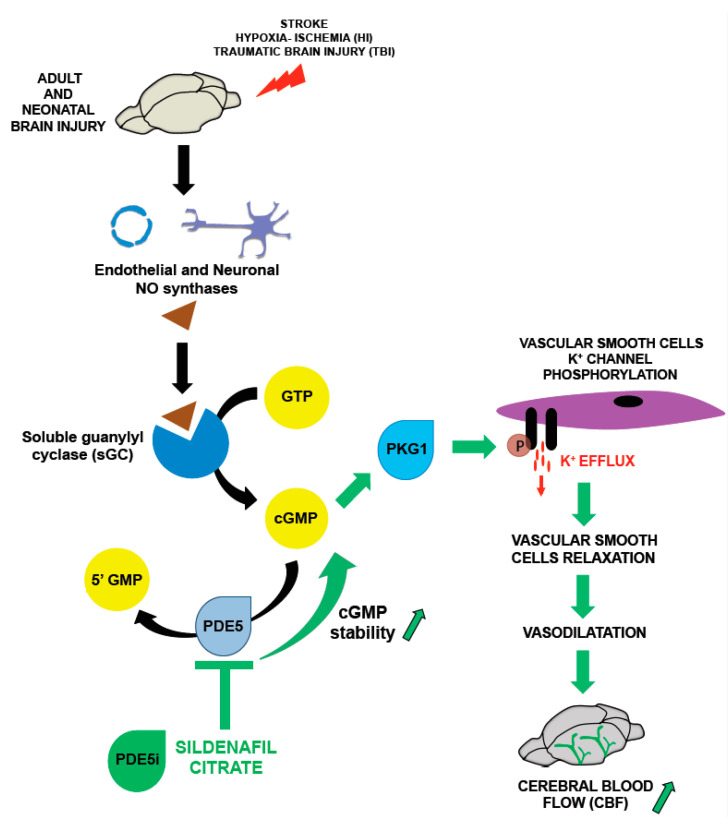
Sildenafil and the modulation of cerebral blood flow (CBF): the binding of NO to sGC, the main NO physiological receptor, stimulated the synthesis of cGMP, which binds to and activates cGMP-dependent protein kinase I (PKG1). Activated PKG1 then phosphorylates the alpha subunit of the vascular smooth cell K^+^ channel, resulting in a K^+^ efflux, cell hyperpolarization, vascular smooth cell relaxation and in an increase of CBF. Sildenafil modulate cGMP stability via inhibition of the enzyme PDE5 that catalyzes the conversion of cGMP into the 5′ GMP inactive form.

Sildenafil, a highly potent selective inhibitor of PDE5, was originally developed for the treatment of hypertension and angina pectoris and is currently approved by the FDA for the treatment of erectile dysfunction and primary pulmonary hypertension [24,25]. Sildenafil is a blood–brain barrier permeable drug [26] and the interest in its use for the treatment of brain diseases characterized by alterations of CBF originated from preclinical studies performed in mice and rabbits that showed a protective role of sildenafil against ischemia-reperfusion injury in the heart [27,28,29] (Figure 1).

In adult rats, a single injection of sildenafil (10 mg/kg) was reported to induce an 8% increase in CBF six weeks after permanent middle cerebral arterial occlusion (MCAO) [30]. Increased CBF in adult rats six weeks after MCAO was also reported after chronic treatment with sildenafil (10 mg/kg/d for 7 days) in two other studies [31,32]. 

In the developing rodent brain, we previously demonstrated that a single intraperitoneal injection of sildenafil at a dose of 10 mg/kg promoted CBF in a rat model of neonatal hypoxia-ischemia by increasing arterial blood flow velocity (BFV) in the basilar trunk (BT) and the contralateral internal carotid (ICA) one hour after drug injection [33].

## 4. Other Effects of Sildenafil in the Brain

The pathogenesis of brain injury is usually characterized by activation of an abnormal inflammatory response, apoptotic and necrotic processes, and the release/accumulation of reactive oxygen species and other excitotoxic molecules. 

**Figure 2:**
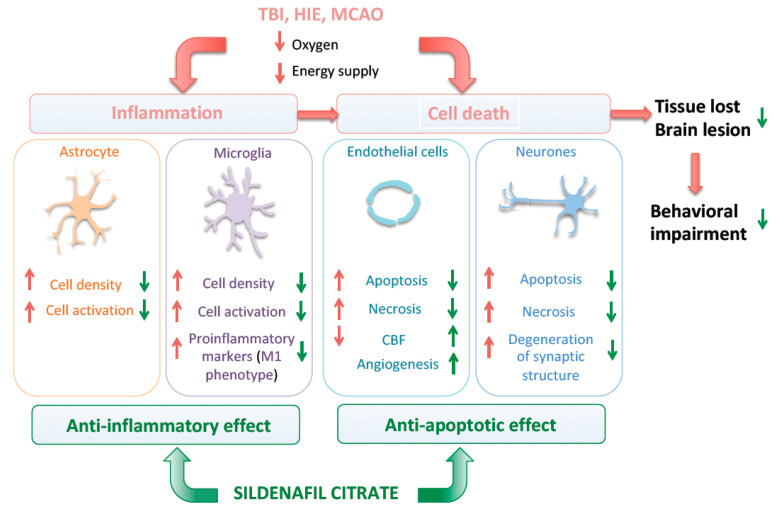
Cellular mechanism associated with the neuroprotective effect of sildenafil.

We summarize here evidence supporting the ability of sildenafil to target and modulate pathological processes other than CBF (Figure 2). In addition to modulation of CBF, the main effects of sildenafil in animal models of brain injury in adults and neonatal rodents are summarized in the Table 1. 

### 4.1. Angiogenesis

Aside from its effects on vascular reactivity, sildenafil has also been shown to be a key modulator of angiogenesis.

Endogenous NO and NO donors promote endothelial cell (EC) migration and growth and the formation of tube-like structures in vitro, as well as angiogenesis in vivo [34,35,36,37,38]. A pro-angiogenic effect of sildenafil was recently demonstrated combining in vivo and in vitro studies in chicken chorioallantoic membranes (CAMs) and endothelial cells. In particular, Pyriochou et al. showed that 48 h of treatment with sildenafil significantly increased the length of vessels in CAMs and stimulated EC migration and their organization into vessel-like structures [39]. 

Histological analysis of MCAO rodent models for both aged and juvenile rats showed sildenafil treatment to increase the number of blood vessels and proliferating endothelial cells in the peri-ischemic region one week after surgery [40,41]. Several studies confirmed the increase of angiogenesis by MRI [30,32] and a similar angiogenic effect was observed in cryolesioned mice three days after injury [42]. 

In a neonatal rat HIE model, sildenafil treatment (10 mg/kg) reduced microvessel damage, as shown by a significant reduction in the number of TUNEL-positive endothelial cells at day 3 post HI. Furthermore, the authors showed protection of the integrity of the blood–brain barrier, shown by a reduction in IgG extravasation in treated animals [33]. The effect of sildenafil administration on angiogenesis in a P10 neonatal rat HIE model is a subject of debate [33,43]. Indeed, oral administration of sildenafil for seven days at a dose comparable to that used by Charriaut-Marlangue et al. had no effect on microvessel density, whereas a significant decrease was reported in response to treatment with 50 mg/kg 20 days after injury [43]. This discrepancy concerning the long-term use of sildenafil with the results of previous adult and neonatal studies was suggested by Yazdani et al. [43] to be the consequence of the physiologically regenerative angiogenesis observed in response to HI, even in the absence of treatment [44,45,46], which hide the earlier angiogenic effect of sildenafil. 

### 4.2. Neuro-Inflammation

The brain inflammatory response is regulated by a crosstalk between microglia and astrocytes [47]. A change in microglia and astrocyte reactivity has been reported to be an important component in the pathogenesis of brain injury disease [48,49].

Cold-light photothrombotic occlusion in the cortical vasculature was found to be associated with an increase in the density of microglia in the ipsilateral hemisphere in adult mice and rats from day 8 to day 28 post stroke [50,51]. Strong immunoreactivity for the specific astrocytic marker glial fibrillary acidic protein (GFAP) was observed by Patience et al. on the ipsilateral side of the brains of adult mice in comparison to the sham animals in the same model [52]. Two other studies performed using the adult MCAO mouse model reported the presence of activated amoeboid microglia and active microglial proliferation in the peri-infarct area [53,54]. Similarly, a significant and prolonged increase in GFAP protein and mRNA levels was reported in a cortical stroke model in response to mild and severe ischemia in rats [55]. In accordance with these observations, increased expression of pro-inflammatory markers (e.g., tumor necrosis factor alpha (TNFα) and Interleukin-1β (IL-1β)) has been described in both animals and humans after an ischemic stroke [56,57,58,59]. 

Consistent with the anti-inflammatory action of sildenafil in HI, a single subcutaneous injection of sildenafil (10 mg/kg) in adult mice, before and 24 and 48 h after the induction of a cortical cryolesion significantly reduced the density of activated microglia and increased astrocyte reactivity three days after injury [42]. In another study, chronic sildenafil treatment (2 mg/kg/day) reduced the expression of the inflammatory markers monocyte chemoattractant protein-1 (MCOP-1) and IL-1β in P28 rats subjected to multiple microinfarction [60].

In neonatal pups, an increase in microglia cell density and activation in the penumbra, in addition to a strong astrocytic response, was described, both in a permanent MCAO mouse model and a rat model of HIE, three days and one week after injury [33,61]. In these models, a single intraperitoneal administration of sildenafil (10 mg/kg) was found to reduce the density and activation of microglia in the penumbra three and seven days following MCAO [33,61]. Furthermore, Moretti et al. showed that sildenafil induces a shift in microglia polarization toward an M2 anti-inflammatory phenotype seven days after brain injury [61]. Interestingly, the upregulation of astrocyte reactivity in the areas of the peri-lesion site three days after the induction of stroke was found to be associated with changes in CBF in response to MCAO [61]. 

### 4.3. Cell Death

Neuronal cell death is the result of multiple mechanisms (e.g., excitotoxic cell death, necrosis, and apoptosis) activated by the reduction of oxygen and energy supply [62]. Neuronal loss has been unanimously reported in animal models of brain injury related to stroke, hypoxia and TBI and the magnitude of tissue loss has been reported to be an important variable in functional recovery [43,62,63].

Pre-clinical studies performed in rodents have highlighted the ability of sildenafil to reduce lesion size in both the adult and developing brain. Moretti and Charriaut-Marlangue demonstrated that a single intraperitoneal administration of sildenafil (10 mg/kg) after surgery can significantly reduce the percentage of tissue loss one week after the injury in two independent studies on mouse and rat models of neonatal stroke and HI [33,61]. Similarly, the oral administration of sildenafil (10 mg/kg/d) for one week to neonatal rats was reported to significantly increase the volume of the ipsilateral hemisphere three weeks after HI [43]. Studies performed in adult models further confirmed the results observed in pups. Sildenafil administration was indeed reported to reduce the number of degenerating neurons and increase the number of surviving cortical and striatum neurons in MCAO rats. Interestingly this pro-survival effect was associated with reduced degeneration of the synaptic structure and an improvement in behavioral performance [63]. The reduction of cell death induced by sildenafil has been described both in mice with cortical cryolesions (reduction in TUNEL-positive cell density) and those with chemically-induced cerebral ischemia (increased expression of the anti-apoptotic proteins Bcl-2 and Bcl-xl) [42,64]. 

The effect of sildenafil on cell death in the context of TBI has been investigated little. However, a recent study performed in a rat TBI model confirmed the neuroprotective effect of sildenafil, with a decrease in neuronal cell death, as shown by a reduction in the density of neurons with pyknotic nuclei in the prefrontal cortex and hippocampus [65].

### 4.4. Neurogenesis

An increase in neurogenesis in the subventricular zone to promote the migration of newborn neurons into the ischemic boundary area is among the mechanisms involved in the normal repair process widely described in adult rat models of ischemic stroke [66,67,68,69]. Emerging evidence suggests that sildenafil could contribute to neuroprotection by increasing neurogenesis. Sildenafil has been shown to increase the number of immature and mature neurons in the ischemic brain when administrated for seven consecutive days to juvenile and adult animals following middle cerebral artery occlusion [70,71]. In addition, an increase in the neuronal density within the boundary of the ischemic core has been observed following sildenafil treatment three weeks after neonatal HI in rats [43].

## 5. Sildenafil Exposure and Behavioral Outcomes in Response to Brain Injury

Neurological sequelae are a common pathological outcome of stroke and hypoxic ischemic encephalopathy (HIE) in the neonate and of stroke and TBI in adult. Cognitive disabilities including memory deficit and language deficits are indeed reported in the 60% of children with perinatal stroke and are identified in the 30% of adult stroke patients within the first year after the brain injury event [72,73]. Similarly cognitive and behavioral alterations are reported in the 30% of patients surviving to TBI [74]. 

Birth complications are considered as an important risk factor for insurgence of the cerebral palsy (CP), a severe condition of motor disabilities observed in children [75]. In agreement with this observation, clinical data identified perinatal stroke as a causal factor in the 50% of the infants born at term and affected by a hemiplegic CP [76,77].

Pre-clinical studies performed on neonatal and adult animal models evidenced the ability of sildenafil treatment to modulate and reverse the brain injury-induced behavioral alterations. A single intraperitoneal administration of sildenafil (10 mg/kg) after surgery is able to mitigate the altered exploratory behavior and general activity observed in response to a neonatal HI event in mice, seven days after surgery [33]. 

Studies performed in adult model confirmed the results obtained in pups. Seven days administration of sildenafil (intravenous 16 mg/kg) significantly improved the motor coordination performance in a rat adult model of MCAO [63]. Similarly, Zhang et al. demonstrated in two independent studies the beneficial effect of a chronic sildenafil treatment on the neurological score and the motor performance [40,70]. 

## 6. Sex Difference in Brain Injury Outcome

Sex is widely recognized as a factor influencing the clinical outcome in response to HI, stroke and TBI in both neonatal and adult ages. 

Neonatal brain injury is clinically associated to a worse outcome in male infants and a higher mortality rate has been reported in male in comparison to female [78,79,80,81]. Similarly, disorientation and loss of consciousness following TBI in human adults were found to be more frequent in men in comparison to women [82,83]. 

Pre-clinical studies designed to investigate the underlining mechanism of this sex-dependent vulnerability of the brain reported conflicting results. The severity of the neurological outcome has been described to be dependent on the developmental stage at the time of injury. HI induced in P3 rats was associated to a greater cognitive impairment and to a greater white matter histological damage in females than in males [84]. In contrast, a higher mortality rate, a higher cognitive impairment and a greater injury severity was reported in P10 male rats [85]. Similarly, an greater vulnerability was observed in male mice exposed to hypoxia from P3 to P11 with an increased hippocampal tissue loss and white matter damage in comparison to female [86]. 

Studies performed on an animal model of TBI highlighted a complex interaction between sex and injury outcomes. O’ Connor et al. evidenced a better behavioral performance in cognitive task in female compared to male [87] whereas, in two other studies, a greater mortality rate was observed in female rats [88] or no change in brain damage was reported between female and male after TBI [89].

The role of sexual hormones progesterone and estrogen as neuroprotectants is supported by preclinical studies showing that ovariectomy exacerbated brain injury [90,91]. However, sex difference in the outcome of brain injury appears to be very complex and is not only limited to the different level of sexual hormones between male and female. As recently reviewed by Charriaut-Marlangue et al. [92] and by Murden et al. [81], differences in cell death pathways, exacerbated microglia activation, and abnormal response to oxidative stress in males are suggested to play a key role.

The effect of sex on sildenafil mediated neuroprotection remains unknown and only one study performed in P9 pups mice evidenced that inhaled nitric oxide reduces brain injury three days after HI, only in males [13].

## 7. Other PDE5 Inhibitors as Potential Neuroprotectors in Preclinical Models

In addition to the neuroprotective role of sildenafil reviewed above, other selective PDE5is have been tested in preclinical models of brain injury [93]. 

Tadalafil is a long-lasting PDE5i with a rapid onset of action and several studies have shown its ability to modulate inflammation, neurogenesis, and cell death in response to prenatal hypoxic brain injury and to cerebral ischemia and stroke in adults [94,95,96,97,98,99]. A recent study performed by Tachibana et al. showed that three days (from E14 to E17) in utero administration of tadalafil is neuroprotective in a mouse model of induced preeclampsia with fetal growth restriction (FGR). In particular, the authors demonstrated that the increased astrocyte cell density observed in the corpus callosum of FGR mice was significantly reduced and synaptogenesis and myelination significantly increased in tadalafil-treated animals at P15 and P30 [94]. The protective effect observed in neonatal models was further confirmed by other studies performed in adult rodent models of cerebral HI. Zhang et al. showed oral administration of tadalafil (2 and 10 mg/kg administrated every 48 h, starting 24 h after the insult) to significantly increase angiogenesis and endothelial cell proliferation in the peri-infarct area and improve neurological recovery in embolic stroked animals [97]. An improvement of cognitive and motor ability associated with a reduction in the size of the brain lesion was also observed in response to pretreatment with tadalafil in a mouse model of ischemia and reperfusion injury [96,98]. Moreover, seven days of oral administration of tadalafil was associated with significantly reduced CA1 hippocampal neuronal cell death and improved short-term memory in an adult gerbil model of transient global ischemia [99].

Finally, a limited number of studies is available on the neuroprotective effect of two others PDE5is: zaprinast and yonkenafil [100,101]. In particular, zaprinast, known for its specific PDE5i activity, has also been shown to be a modulator of peroxisome proliferator-activated receptor gamma (PPARγ) and an agonist of G-protein coupled receptor 35 (GPR35), which is involved in the modulation of inflammatory pain [102,103,104]. The two drugs were investigated by Gao et al. and Chen et al. in a rat model of MCAO occlusion. Zaprinast treatment significantly increased CBF and reduced the size of brain lesions when administrated intravenously at a dose of 10 mg/kg 10 min after permanent MCAO [100]. The study performed by Chen et al. in a model of ischemia and reperfusion injury showed that yonkenafil treatment significantly reduced lesion size and neuronal cell death in the cortex and striatum. The reduced neuronal loss was associated with an improvement of neurological outcome and a reduction in the loss of synapses in both the cortex and striatum [101]. 

## 8. Main Human Brain Injuries in Adults and Neonates as Potential Targets for Sildenafil

### 8.1. Adult Brain Injury

Ischemic stroke and TBI are two major causes of disability and mortality in adults. Their worldwide incidence has been steadily increasing and their annual incidence in Europe is 7–8/100,000 [105] and 10–12/100,000 [106], respectively. 

Vascular risk factors, such as hypertension, hypercholesterolemia, diabetes, obesity, and smoking, have been identified as a major cause in the increasing incidence of stroke [107]. Depending on the severity of the injury and the duration of CBF reduction and subsequent oxygen and glucose deprivation, ischemic stroke can be classified as focal or global and transient or permanent [108]. Three different areas are defined on the basis of the morphological, biochemical, and structural alterations observed: (i) core, (ii) penumbra, and (iii) benign oligemia. The core region corresponds to the core of the stroke and represents the tissue that is irreversibly compromised, whereas the penumbra and benign oligemia identify the tissue surrounding the core area that is still viable and potentially recoverable, either spontaneously or by therapeutic treatment, including sildenafil [109]. 

TBI is generally the consequence of an external mechanical insult and its pathophysiology is defined by primary and secondary brain injury [110]. Primary injury is linked to the mechanical insult and is clinically identified by the presence of skull fracture, coup and contrecoup contusions and cerebral hemorrhage. The outcome of the functional and structural alterations observed in the primary injury promote the appearance of secondary injury, defined by the development of edema, compression of the cerebral tissues, an increase in intracranial pressure, a decrease in CBF, and the appearance of ischemic brain damage, the latter identified in more than 90% of TBI patients [110,111,112,113]. Alterations in CBF induced by TBI are characterized by an initial large decrease, followed by an increase, and studies confirmed the association between CBF and poor outcomes for patients with severe TBI [114,115,116]. 

### 8.2. Neonatal Brain Injury

In term-neonates, stroke and hypoxic ischemic encephalopathy (HIE) related to perinatal asphyxia are two major causes of perinatal death and long-term neurological disabilities, including cerebral palsy, mental retardation, and epilepsy [117,118].

Neonatal stroke is clinically defined as cerebrovascular events that occur between week 20 of gestation and the first 28 postnatal days [119]. Epidemiological data have shown an annual incidence of 1/2500 to 1/5000 and an annual mortality rate estimated to be 3.5/100,000 [119,120,121,122]. Although perinatal stroke affects both preterm and term neonates, the brain injury profile reflects the maturational stage of the brain at the time of the stroke. Exposure to stroke in preterm babies occurs during a critical period for the differentiation of oligodendrocyte progenitors (OPC) to mature myelinating neural cells, a process disrupted by microglial activation and neuro-inflammation. Due to such a temporal overlap, neonatal stroke in infants born preterm has been shown to be a strong predisposing factor for periventricular white-matter injury and cerebral palsy [123,124,125]. Conversely, a lesion observed in the gray matter, mainly in the cortex, thalamus, and striatum, has been reported for term infants in response to stroke [120].

In addition, poor perfusion of the placenta, preeclampsia, and chorioamnionitis are clinically associated with a reduction in fetal CBF and a risk of neonatal stroke [126,127,128]. 

HIE is a major complication commonly observed in term neonates following perinatal asphyxia leading to high incidence of neonatal death and long-term neurodevelopmental impairment [117]. Its incidence has been recently estimated to be 1.5 per 1000 live births in developed countries and between 2.3 and 26.5 per 1000 live births in developing countries [117,129]. The exact event linked to HIE in term neonates is sometimes unknown. However, the clinical history of infants affected by HIE showed cord prolapse, uterine rupture, placenta praevia, and maternal hypotension to be important causal factors [117]. 

Reduced CBF and the subsequent reduction in the supply of oxygen to the brain are the two key elements in the pathophysiology of HIE and stroke in neonates responsible for the activation of a cascade of processes and cellular mechanisms, identified as primary and secondary energy failure [117,130,131]. Primary energy failure in cerebral stroke and HIE is the direct consequence of reduced CBF and oxygen and glucose deprivation, leading to a major challenge to cell integrity. Normal cellular physiology and the biological processes involved in the maintenance of cell homeostasis are disrupted, with the consequent release of glutamate, increase in intracellular calcium and sodium, and activation of necrotic and apoptotic processes. Secondary energy failure, well documented following HIE, occurs between 6 and 48 h after the initial injury and is associated with the detrimental consequences of an abnormal inflammatory response, the accumulation of reactive oxygen species and the release of excitotoxic molecules [117]. Many of these biological responses to perinatal asphyxia are potential targets of sildenafil. 

## 9. Future Perspectives

Based on preclinical observations (Table 1), sildenafil has been tested in human clinical trials to evaluate its efficacy in the treatment of adult brain diseases and injury characterized by alterations in cerebral hemodynamic function (Table 2). 

Some trials have not yet been completed and some of them have been interrupted due to slow recruitment. However, a recent study performed on patients with chronic TBI demonstrated an improvement in vascular functionality in response to drug treatment [132]. A single oral administration of sildenafil (50 mg) was associated with an increase in and normalization of cerebrovascular reactivity in adult TBI subjects [132].

The studies discussed in this review strongly suggest a neuroprotective role for sildenafil. Originally conceived as a PDE5 inhibitor for the treatment of cardiac and pulmonary dysfunction, sildenafil can modulate the complex and multiple pathogenic processes underlying the damage induced by stroke, HIE, and TBI brain injury. 

In this context, the association of sildenafil with other therapeutic strategies could represent a good option to improve recovery after brain injury in both adults and neonates. A clinical trial is currently ongoing to evaluate the potential effect of the association of oral sildenafil and hypothermia, the only currently available therapy to treat term asphyxiated newborns (Sildenafil Administration to Treat Neonatal Encephalopathy (SANE) and Repair Brain Injury Secondary to Birth Asphyxia: A Randomized, Double-blind, Placebo-controlled Pilot Phase Ib Study; NCT02812433 and NCT04169191). Another multicenter randomized clinical trial is planned to start in 2022 to test the added value of parenteral sildenafil combined with hypothermia to prevent brain lesions following birth asphyxia (Shine trial).

## Figures and Tables

**Table 1:** Main effects of sildenafil according to preclinical models of brain injury from neonatal to adult rodents. HI: hypoxia-ischemia; TBI/Cryo: traumatic brain injury/cryolesion.

Effect	Neonatal	Juvenile	Adult	
	HI			TBI/Cryo
CBF	+++	+	+	?
Angiogenesis	+/−?	+	+	+
Inflammation	+++			
Cell death	++	++	++	+
Neurogenesis	+	++	++	?

**Table 2:** Ongoing or completed clinical trials testing sildenafil to prevent brain injury.

NCT/Status/Study Title	Condition	Comparator	Phase	Primary Outcome	Main Effect	Comment/Reference
NCT02628847 (Terminated) Sildenafil and Stroke Recovery	Adult stroke	Placebo	1	Motor recovery at one and three months	Unknown	Recruitment was problematic
NCT00452582 (Terminated) Sildenafil (Viagra) Treatment of Subacute Ischemic Stroke	Adult ischemic stroke	Usual care	1	The maximum tolerated dose and toxicity profile of sildenafil treatment in patients with subacute ischemic stroke	Sildenafil appeared to be safe in patients with mild to moderately severe stroke	Failure to recruit in expected time period
NCT03855332 (Recruiting) Oxford Haemodynamic Adaptation to Reduce Pulsatility Trial (OxHARP)	Small vessel cerebrovascular disease in adult	Cilostazol Placebo	2	Middle cerebral arterial pulsatility index	-	Estimated study completion date: December 2022
NCT01762475 (Completed) Sildenafil for Cerebro-vascular Dysfunction in Chronic Traumatic Brain Injury	Traumatic brain injury in adults	-	2	Cerebrovascular reactivity	Single-dose sildenafil improves regional CVR deficits in chronic TBI patients	[116]
NCT02114775 (Completed) Growth Hormone or Sildenafil as Therapies for Fatigue in Mild-Traumatic-brain-injury (MTBI)	Traumatic brain injury in adults	Genotropin Placebo	1	Performance fatigue	Unknown	Sildenafil was found to increase protein synthesis and reduces muscle fatigue in healthy men
NCT04058132 (Recruiting) Cerebrovascular Reactivity Assessed With fNIRS as a Biomarker of TCVI After Acute Traumatic Brain Injury in Military	Acute/subacute traumatic brain injury in adults	None	2	Variation of oxyhemoglobin and deoxyhemoglobin concentration Longitudinal measure of CVR	-	Estimated study completion date: April 2021
NCT02990078 (Recruiting) Non-invasive Measurement of Cerebrovascular Reactivity After Traumatic Brain Injury	Traumatic brain injury in adults	None	1	Change in CVR	-	Estimated study completion date: December 2026
NCT03417492 (Recruiting) Cerebrovascular Reactivity in American Football Players	Traumatic brain injury in adults	None	1	Effect of single dose sildenafil citrate on global BOLD response to hypercapnia	-	Estimated study completion date: September 2022
NCT02812433 (Active) Sildenafil Administration to Treat Neonatal Encephalopathy	Hypoxic ischemic encephalopathy in neonates	Ora-Blend	1	Serious adverse events	-	Estimated study completion date: June 2022

## Data Availability

Not Applicable.
